# Guided growth with tension band plate or definitive epiphysiodesis for treatment of limb length discrepancy?

**DOI:** 10.1186/s13018-019-1139-4

**Published:** 2019-04-11

**Authors:** Paul Borbas, Christoph A. Agten, Andrea B. Rosskopf, Andreas Hingsammer, Karim Eid, Leonhard E. Ramseier

**Affiliations:** 1 Department of Orthopaedics, Kantonsspital Baden, Baden, Switzerland; 2 Department of Orthopaedics, Balgrist University Hospital, Zurich, Switzerland; 3 Department of Radiology, Balgrist University Hospital, Zurich, Switzerland

**Keywords:** Epiphysiodesis, Leg length discrepancy, Limb length, Leg length correction

## Abstract

**Background:**

It is not exactly known whether guided growth or definitive epiphysiodesis techniques are superior in treating limb length discrepancy (LLD). The purpose of the present study was therefore to find out if definitive epiphysiodesis is associated with more powerful LLD correction than tension band plate epiphysiodesis.

**Methods:**

Pediatric patients with LLD treated either with tension band plating as a guided growth technique (temporary epiphysiodesis) or a percutaneous drilling technique (definitive epiphysiodesis) around the knee and a minimum follow-up of 12 months were included in this retrospective study. Radiographic measurements were performed by two independent reviewers. The reduction in side difference between preoperative radiographs and last follow-up was calculated and compared between surgical techniques.

**Results:**

Thirty-eight patients (mean age 13.6 years) were included, 17 treated with temporary and 21 with definitive epiphysiodesis. Average follow-up was at 578 days. The reduction of the LLD in 12 months was 5.7 mm in patients treated with temporary epiphysiodesis and 8.4 mm with definitive epiphysiodesis, respectively (*p* = 0.22). In both groups, LLD could be statistically significantly reduced after 12 and 24 months. Definitive epiphysiodesis had a lower revision rate (4.8% vs. 17.6%). Intra- and interobserver reliability of the measurements was excellent.

**Conclusions:**

As in earlier studies supposed, temporary epiphysiodesis with tension band plating seems to correct LLD less powerful compared to definitive percutaneous epiphysiodesis. However, in the present study, the differences of LLD correction were not statistically significant. We do not recommend the use of tension band plates for LLD correction due to inferior correction with higher complication and revision rate.

## Background

In children with open epiphyses and predicted leg length discrepancy (LLD) at maturity between 2 and 5 cm, epiphysiodesis is the treatment of choice [1]. Phemister was the first who published an open fusion technique of the growth plate [2]. Over the years, less invasive techniques for irreversible growth arrest have been described and are most commonly performed percutaneously [3, 4]. Potentially reversible epiphysiodesis techniques became more and more popular [5, 6]. A guided growth technique with tension band plates—so-called eight-plates—first described by Stevens et al. for angular correction received also popularity for correction of LLD [7]. Effective correction of LLD with tension band plates has been reported in two studies [8, 9].

Recently, however, the efficiency of epiphysiodesis for LLD correction with tension band plates was doubted and irreversible epiphysiodesis was favored in several studies [10–12].

The purpose of the present study was therefore to find out if definitive percutaneous epiphysiodesis is superior to tension band plate epiphysiodesis for LLD correction.

## Methods

The study was approved by the institutional review board and by the local ethics committee.

Data from all patients undergoing epiphysiodesis for correction of LLD between January 2006 and December 2012 was collected retrospectively. Study inclusion criteria were LLD correction with epiphysiodesis and a minimum follow-up of 12 months after surgery. Patients with additional correction of angular deformities, skeletal dysplasia, malignancy, Blount disease, or follow-up less than 1 year were excluded.

Skeletal age was analyzed according to the method of Greulich and Pyle [13]. Expected LLD at maturity had to be at least 2 cm as an indication for surgery.

### Surgical technique

**Fig. 1:**
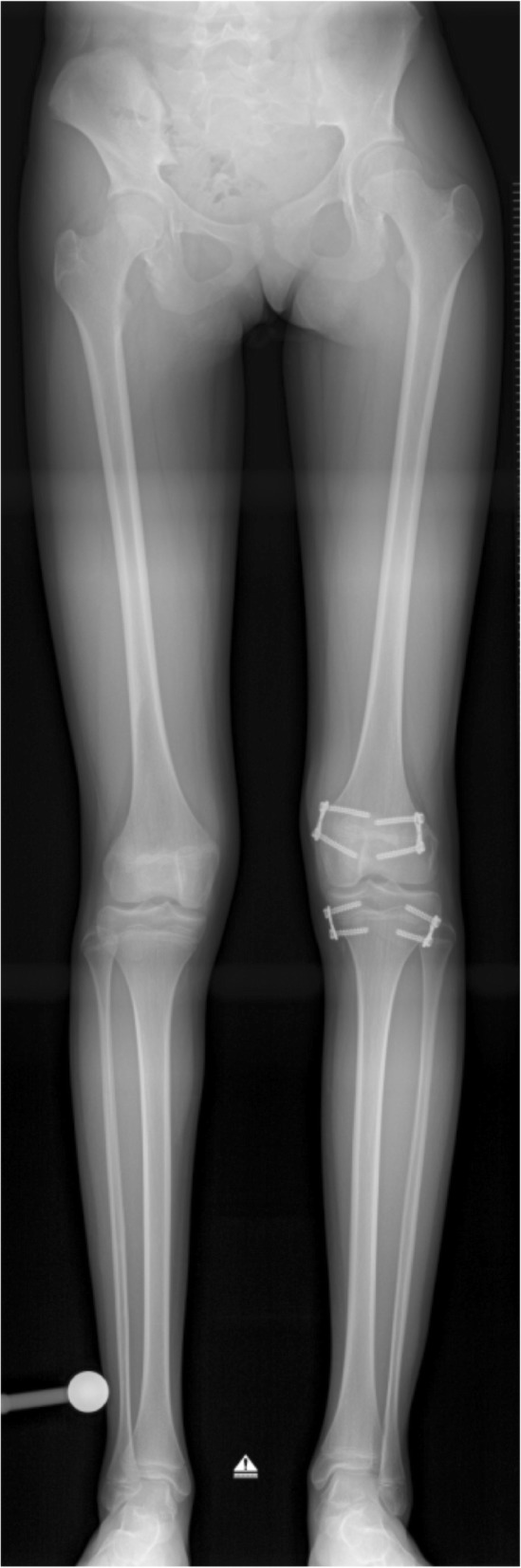
Long-standing X-ray of a patient with limb length discrepancy. Temporary epiphysiodesis with eight-plates (TE) was performed as a tension band procedure with one plate at both the medial and lateral side of the epiphysis of the left distal femur and proximal tibia

Temporary epiphysiodesis with eight-plates (TE) was performed as a tension-band procedure with one plate at both the medial and lateral side of the epiphysis (Fig. 1).

In all patients treated with TE, metal removal was routinely performed either after desired leg length was reached or after growth completion.

Definitive epiphysiodesis (DE) was performed in a modified Canale technique with disruption of the growth plate from both sides with the aid of drills and an angulated curette [4]. Both techniques were performed under fluoroscopic control. Surgery was always performed around the knee—in the distal femur alone, the proximal tibia alone, or in both.

Postoperatively, full weight bearing as tolerated on crutches was allowed immediately in both groups.

### Radiographic assessment

Measurements were performed by two independent reviewers.

**Fig. 2:**
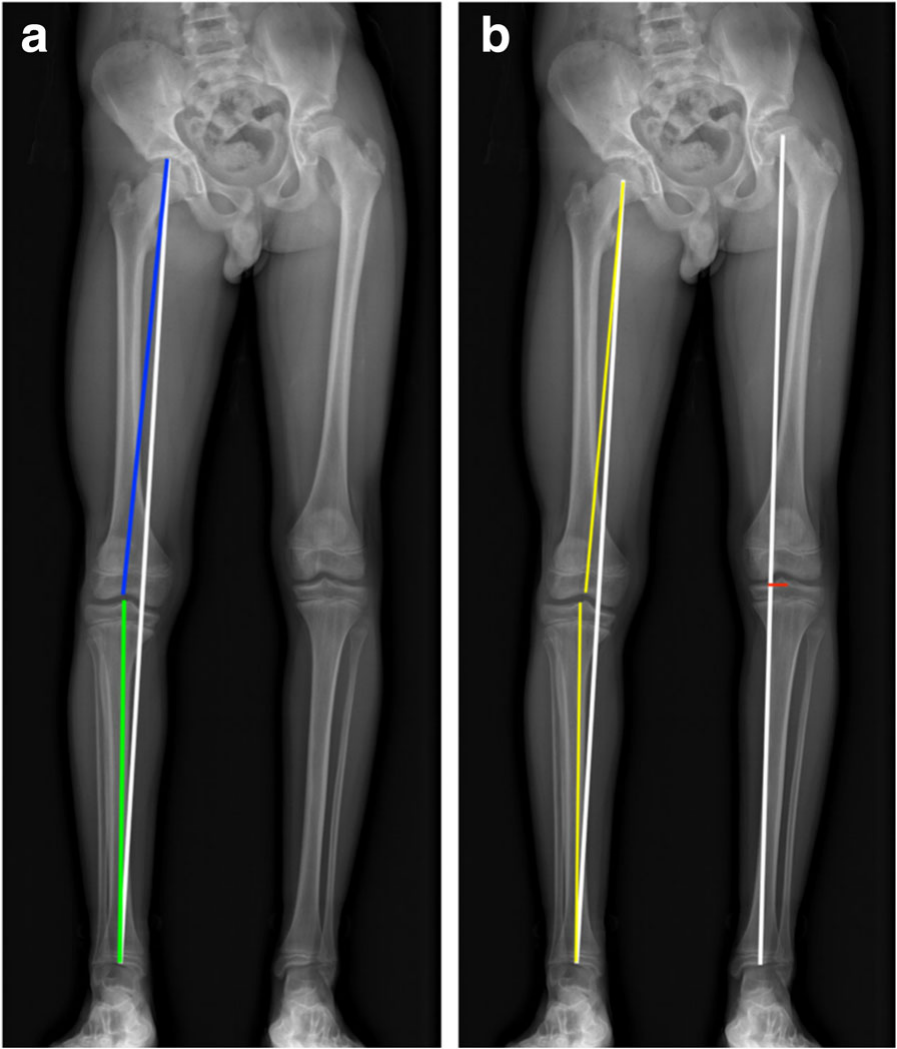
Measurements were performed on long-standing X-rays. **a** Limb length (white), femoral length (blue), tibial length (green). **b** Mechanical axis (white), mechanical axis deviation [MAD] (red), axial deviation (yellow)

The detailed distances and angles are shown in Fig. 2. All measurements were performed in both legs on standardized long-standing X-rays with the patellae directed forward.

Measurements were performed on long-standing X-rays preoperatively, after 6 weeks, 1 year, and 2 years postoperatively. LLD difference as well as difference of femur and tibia length only was measured at all points in millimeters.

Limb length was measured from the most superior portion of the femoral head to the center of the tibial plafond at the ankle joint. Femoral length was measured from the most superior portion of the femoral head to the most distal part of the intercondylar notch. The tibial length was measured from the tibial spine to the center of the tibial plafond.

Mechanical axis was drawn from the center of the femoral head to the center of the tibial plafond. The mechanical axis deviation (MAD) was measured if the axis did not pass through the center of the knee joint—with positive values for a medial axis in genu varum and negative values for a lateral axis genu valgum as the distance from the tibial spine perpendicular to the tibial plateau in millimeters. The axial deviation was further measured in degrees.

In case of a revision surgery, the last follow-up before revision surgery was included—for example, given in patients with TE who had undergone early metal removal between 12 and 24 months postoperatively, only the follow-up at 12 months was studied.

### Statistical analysis

Statistical analysis was performed using IBM SPSS® statistics software (version 22.0, Armonk, NY). Interrater agreement was calculated using intraclass correlation coefficients (ICC). ICC can have a value between 0 (no agreement) and 1 (absolute agreement) and was classified according to Fleiss as excellent if larger than 0.75 [14].

Paired *t* test was used to evaluate pre- and postoperative values. Group differences were analyzed with chi-square test. Differences of change in the time periods—as LLD change—between DE and PE group were evaluated with unpaired *t* test. *p* values ≤ 0.05 were deemed statistically significant.

## Results

Thirty-eight patients were included in the study, with 21 in the DE group and 17 in the TE group. The average age at surgery was 13.6 years. There were no statistical differences between the groups, apart from the location around the knee where surgery was performed (*p* = 0.021) and sex (*p* = 0.02). Detailed patient demographics are illustrated in Table 1.

**Table 1:** Comparison of temporary and definitive epiphysiodesis group

	Temporary ED	Definitive ED	Comparison of groups (*p* value)
*n*	17	21	
Sex (female/male)	3/14	11/10	0.02*
Side (left/right)	13/4	12/9	0.05
Location (femur/tibia/both)	6/7/4	10/1/10	0.02*
Age (years)	13.4 ± 0.4	13.9 ± 0.2	0.2
Height (cm)	164.2 ± 3.3	165.9 ± 3.8	0.57
Weight (kg)	56.2 ± 3.9	59.9 ± 5	0.74
Preoperative limb length discrepancy (mm)	22.2 ± 1.9	23.2 ± 2.2	0.73
Preoperative femur length discrepancy (mm)	11.8 ± 2.2	15.2 ± 1.9	0.25
Preoperative tibia length discrepancy (mm)	10.3 ± 2.0	7.8 ± 1.8	0.36
Preoperative MAD difference (mm)	5.1 ± 2.4	1.1 ± 2.5	0.27
Preoperative axial deviation difference (°)	2.1 ± 1.0	0.2 ± 0.7	0.13

*ED* epiphysiodesis, *LLD* limb length discrepancy, *MAD* mechanical axis deviation

*Significant difference

Etiologic factors of LLD are listed in Table 2.

**Table 2:** Etiologies of limb length discrepancy

	Temporary ED	Definitive ED
Idiopathic/congenital	5 (29%)	6 (29%)
Posttraumatic	2 (12%)	5 (24%)
Perthes disease	3 (18%)	2 (10%)
Postsurgical	2 (12%)	2 (10%)
Clubfoot	4 (24%)	0
DDH	1 (6%)	1 (5%)
Hemihyperthrophy or vascular malformation	0	3 (14%)
Morbus Trevor	0	1 (5%)
Osteomyelitis	0	1 (5%)

The average final follow-up was at 578 days. The reduction of the LLD in 12 months was 5.7 mm in patients treated with TE and 8.4 mm with DE, respectively. This difference was, however, statistically not significant (*p* = 0.22). The percentage of improvement after 1 year was 26% in the TE group and 36% in the DE group. Detailed results at 1-year follow-up are depicted in Table 3.

**Table 3:** Outcome in temporary and definitive epiphysiodesis group at 1-year follow-up

	Temporary ED	Definitive ED	Comparison of groups (*p* value)
*n*	17	21	
Preoperative limb length discrepancy (mm)	22.2 ± 1.9	23.2 ± 2.2	0.73
Reduction of LLD in 1 year (mm)	5.7 ± 1.6	8.4 ± 1.4	0.22
MAD change in 1 year (mm)	0.3 ± 2.8	0.5 ± 1.4	0.77
Axial deviation change in 1 year (°)	0.2 ± 1.0	0.1 ± 0.4	0.37

In 27 patients, the reduction of LLD was analyzed at 2-year follow-up. Patients treated with TE (*n* = 15) had a reduction of 12.2 mm (55% improvement) and those treated with DE (*n* = 12) of 17.9 mm (77% improvement), respectively. Again, these differences were not statistically significant (*p* = 0.16).

Individual results of tibial and femoral length correction did not show any statistically significant differences at 1- and 2-year follow-up as well (Table 4). Interestingly, after 2 years, tibial length correction was better in the TE group than in the DE group (*p* > 0.05).

**Table 4:** Individual outcome in temporary and definitive epiphysiodesis group

	Temporary ED	Definitive ED	Comparison of groups (*p* value)
Reduction of tibia length discrepancy in 1 year (mm)	3.2 ± 1.8 (*n* = 11)	3.2 ± 1.2 (*n* = 11)	0.9
Reduction of tibia length discrepancy in 2 years (mm)	5.4 ± 2.5 (*n* = 11)	4.7 ± 2.3 (*n* = 8)	0.83
Reduction of femur length discrepancy in 1 year (mm)	4.3 ± 1.6 (*n* = 10)	6.2 ± 0.8 (*n* = 20)	0.25
Reduction of femur length discrepancy in 2 years (mm)	10.1 ± 3.0 (*n* = 9)	12.1 ± 1.7 (*n* = 12)	0.57
Reduction of LLD in 1 year (mm)	5.7 ± 1.6 (*n* = 17)	8.4 ± 1.4 (*n* = 21)	0.22
Reduction of LLD in 2 years (mm)	12.2 ± 2.7 (*n* = 15)	17.9 ± 3.0 (*n* = 12)	0.16

In both groups, LLD was successfully reduced after 12 and 24 months (*p* < 0.001). No difference could be seen in axial alignment and MAD change. Intra- and interobserver reliability of the measurements was excellent (ICC = 0.9).

### Complications

DE had a lower revision rate compared to TE (4.8% vs. 17.6%).

**Fig. 3:**
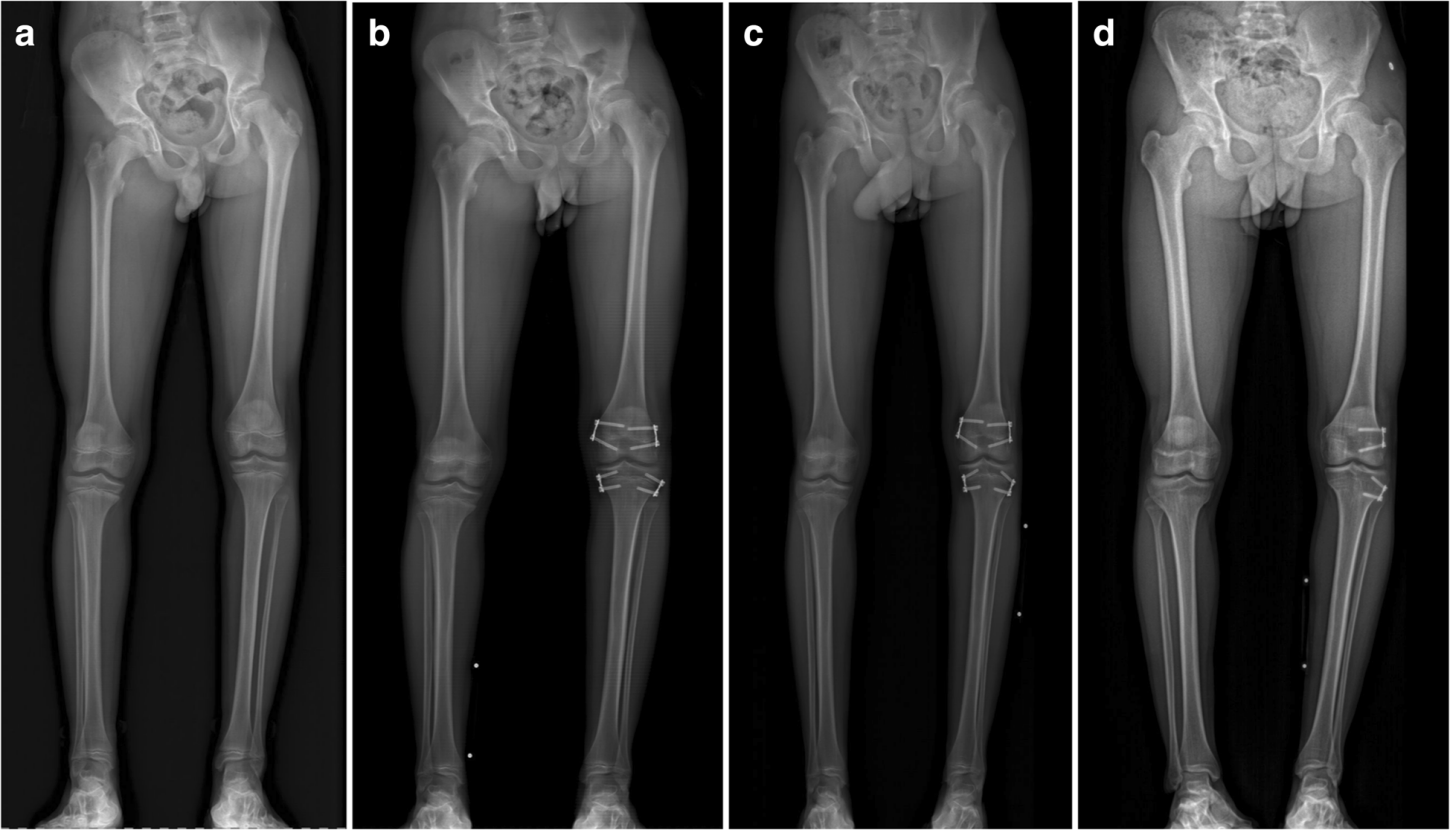
Case of a 14-year-old male patient with idiopathic limb length discrepancy. **a** Preoperative X-ray, demonstrating pelvic obliquity and shortening of the right leg of 3.9 cm. **b** Postoperative X-ray after the patient was treated with tension band plates on both medial and lateral epiphyses of the distal femur and proximal tibia. **c** At 1-year follow-up, partial metal removal had to be performed due to the progression of varus malalignment. Tension band plates of the medial distal femur and medial proximal tibia were removed. **d** At 2.5-year follow-up, varus malalignment was improved to 6° with a residual LLD of 1.4 cm

In one patient treated with TE, partial metal removal had to be performed due to the beginning of axial malalignment. The patient was initially treated with tension band plates on both medial and lateral epiphyses of the distal femur and proximal tibia. Due to a 5° progression of varus malalignment at the 1-year follow-up, removal of only the metal around the medial distal femur and medial proximal tibia was performed at that time. The result at 2.5 years showed a good correction (Fig. 3).

Another patient, who was treated with TE only around the proximal tibia epiphysis, developed a progression of 4.5° of a valgus malalignment. Because of that, additional tension band plating of the lateral epiphysis of the distal tibia was performed.

Furthermore, in a patient of the TE group, secondary DE of the distal femur epiphysis had to be performed after 1 year. The patient was initially treated with TE only around the proximal tibia epiphysis, but the correction of this procedure alone was not sufficient as there was an increasing femoral length difference.

In one patient, who was initially treated with DE of the distal femur epiphysis only, additional DE of the proximal tibia epiphysis had to be performed due to insufficient LLD correction.

Altogether, in two patients of the TE group (11.7%), a second surgery had to be performed because of malalignment. No patient had that problem in the DE group. In both groups, one patient had to undergo an additional procedure because of insufficient LLD correction (5.9% of TE, 4.8% of DE).

## Discussion

In the present study, both evaluated epiphysiodesis techniques showed sufficient LLD correction. However, as supposed in previous studies, LLD correction with tension band plating seems to be slightly less effective compared to definitive epiphysiodesis. Nevertheless, the differences of 5.7 mm versus 8.4 mm after 1 year and 12.2 mm versus 17.9 mm of LLD correction after 2 years were not statistically significant in the present study. In the individual analyses of tibial or femoral epiphysiodesis, differences are even less distinct (Table 4).

Patients treated with TE had a higher revision rate in our study. Two patients (11.7%) had to undergo a second surgery because of malalignment, whereas no patient had that problem in the DE group. In both groups, one patient underwent secondary DE due to insufficient LLD correction after 1 year (5.9% vs. 4.8%). In these patients, only epiphysiodesis of one epiphysis around the knee was performed initially. As a consequence, additional epiphysiodesis of the untreated epiphysis was necessary to correct LLD.

One further issue worth to discuss is the fact that all patients after TE were scheduled for a second surgery to remove the metal. On the one hand, the rate of additional surgery should be as minimal as possible. On the other hand, the principle of TE as a guided growth technique gives the possibility to react in case of corrected LLD and avoid overcorrection. Furthermore, one can react in cases of malalignment with partial metal removal or additional unilateral tension band plating.

This present study represents one of the largest reported series in the literature comparing TE with DE. However, one weakness is the limited number of patients.

Further limitations of the current study should be acknowledged. The study was retrospective and patients were not randomized or matched. However, only one published study in the literature with a retrospective design as well had a higher number of patients included [8]. That study did not give any results in terms of mechanical axis deviation, though. Bayhan et al. included 24 patients in an eight-plate group (TE) and 48 patients in a percutaneous group (DE). In their study, both groups showed to be effective for LLD correction. However, the percentage of improvement was significantly lower in the eight-plate group with a *p* value of 0.031 (41 vs. 58%), which was calculated as the initial minus final discrepancy, and the result was divided by the initial discrepancy. There was, however, no evaluation of tibial or femoral treatment individually in that assessment. In the individual analysis of femoral and tibial correction, no significant differences were found in that study. In both groups, final LLD below 2 cm was reached, with an average correction of 12 mm in the eight-plate group and 16 mm in the percutaneous epiphysiodesis group. These results are very similar to our results with final LLD correction of 12.2 mm in the TE groups and 17.9 mm in the DE group, respectively.

Some authors of further studies formulated the lower efficiency for LLD correction observed with eight-plate epiphysiodesis more clearly. Gaumétou et al. concluded that growth arrest observed after eight-plate technique was unpredictable and lower than that achieved with percutaneous epiphysiodesis using transphyseal screws (PETS) [10]. However, there was no control group in the study. Thirty-two patients were included and an expected growth arrest was calculated. Tibial efficiency with 42% was lower than femoral efficiency with 68% of expected growth arrest at final follow-up at 18 months.

Stewart et al. published a study on 27 patients with 11 treated with dual eight-plate and 16 treated with a physeal ablation technique, similar to the DE group in the present study [12]. Statistically significant difference was shown with 15.5 mm LLD correction in the ablation group compared to 4 mm in the eight-plate group. However, the study had major limitations that were published in a letter to the editor of Kaymaz et al. [15]. Technical and methodological errors are listed in that article.

A further limitation of the present study was that no individual analysis regarding etiologic factors was performed. However, the etiologies of LLD in both groups are listed in Table 2 and are considerably similar.

As a result of the inferior LLD correction with higher complication and revision rate, we personally do not use tension band plates anymore for that indication in our department, except in very rare cases of combined complex angular and length deformities in young children.

## Conclusions

As reported in earlier studies, temporary epiphysiodesis with tension band plating seems to correct LLD less powerful compared to definitive percutaneous epiphysiodesis. However, in the present study, the differences of LLD correction were not statistically significant. Sufficient LLD correction to an average of below 2 cm could be reached in both groups, though. Definitive epiphysiodesis had a lower revision rate (4.8% vs. 17.6%). We do not recommend the use of tension band plates for LLD correction due to inferior correction with higher complication and revision rate.

## Abbreviations

DE: Definitive epiphysiodesis
LLD: Limb length discrepancy
MAD: Mechanical axis deviation
PETS: Percutaneous epiphysiodesis using transphyseal screws
TE: Temporary epiphysiodesis
